# Epigenetic Dysregulation of Somatostatin Receptors (SSTR) 1–5 and Therapeutic Implications in Neuroendocrine and Non‐Neuroendocrine Malignancies

**DOI:** 10.1002/ijc.70635

**Published:** 2026-07-03

**Authors:** Neeraj Kumari, Pablo Hoffmann, Laura Reichl, Maximilian Schmutz, Nina Fischer, Thi Tuong Vi Dang, Fulvia Ferrazzi, Pascal Johann, Michaela Kuhlen, Constantin Lapa, Rainer Claus

**Affiliations:** ^1^ Pathology, Faculty of Medicine University of Augsburg Augsburg Germany; ^2^ Hematology and Oncology, Faculty of Medicine University of Augsburg Augsburg Germany; ^3^ Comprehensive Cancer Center Augsburg (CCCA), Faculty of Medicine University of Augsburg Augsburg Germany; ^4^ Institute of Pathology Friedrich‐Alexander‐Universität Erlangen‐Nürnberg Erlangen Germany; ^5^ Department of Nephropathology, Institute of Pathology Friedrich‐Alexander‐Universität Erlangen‐Nürnberg Erlangen Germany; ^6^ Comprehensive Cancer Center Erlangen‐EMN (CCC ER‐EMN) Erlangen Germany; ^7^ Bavarian Cancer Research Centre (BZKF) Erlangen Germany; ^8^ Pediatrics and Adolescent Medicine, Faculty of Medicine University of Augsburg Augsburg Germany; ^9^ Nuclear Medicine, Faculty of Medicine University of Augsburg Augsburg Germany; ^10^ Bavarian Cancer Research Center (BZKF) Augsburg Germany

**Keywords:** epigenetic priming, epigenetic regulation, neuroendocrine tumors, radiopharmaceutical therapy, somatostatin receptors, SSTR2 expression

## Abstract

Somatostatin receptors (SSTR) mediate the antiproliferative, antisecretory, and proapoptotic effects of somatostatin and its synthetic analogs. Their surface expression on neuroendocrine tumor (NET) cells is required for somatostatin analog therapy and radiopharmaceutical therapy (RPT). However, 20%–30% of gastroenteropancreatic NETs and most poorly differentiated neuroendocrine carcinomas show insufficient SSTR expression, limiting therapeutic eligibility. Accumulating evidence indicates that reversible epigenetic mechanisms contribute substantially to SSTR loss, alongside tumor dedifferentiation, lineage‐state changes, and clonal evolution with DNA hypermethylation of the *SSTR2* promoter representing a central event, complemented by chromatin remodeling and non‐coding RNA‐mediated regulation. Preclinical studies demonstrate that epigenetic therapies, including DNA methyltransferase and histone deacetylase inhibitors, can restore functional SSTR expression. Notably, the first‐in‐human LANTana trial provides clinical proof‐of‐concept that epigenetic priming with oral decitabine/cedazuridine can induce SSTR2 re‐expression and enable subsequent RPT in receptor‐negative tumors. This review synthesizes current knowledge of epigenetic regulation across all five SSTR subtypes (SSTR1–SSTR5), examines receptor silencing in neuroendocrine and non‐neuroendocrine malignancies, and critically evaluates the preclinical and clinical evidence supporting therapeutic strategies that target these mechanisms.

## Graphical abstract

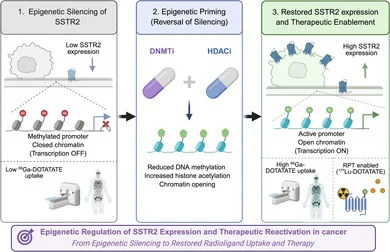

**Abbreviations**
aa: amino acids
AE: adverse event
ARRB2: β‐arrestin 2
AUC: area under the curve
bHLH: basic helix–loop–helix
cAMP: cyclic adenosine monophosphate
CDA: cytidine deaminase
CMTM3: CKLF‐like MARVEL transmembrane domain‐containing protein 3
CpG: Cytosine‐phosphate‐guanine dinucleotide
CRC: colorectal cancer
CRE: cAMP response element
CREB: cAMP response element‐binding protein
D2R: dopamine receptor type 2
DNA: deoxyribonucleic acid
DNMT: DNA methyltransferase
DNMTi: DNA methyltransferase inhibitors
EC50: half maximal effective concentration
EZH2: enhancer of zeste homolog 2
GEP‐NETs: gastroenteropancreatic neuroendocrine tumors
GH: growth hormone
H2AK119: histone H2A lysine 119
H3K27me3: histone H3 lysine 27 trimethylation
H3K4me3: histone H3 lysine 4 trimethylation
H3K9Ac: histone H3 lysine 9 acetylation
HAT: histone acetyltransferase
HDAC: histone deacetylase
HDACi: histone deacetylase inhibitors
HELLS: helicase lymphoid specific
HNSCC: head and neck squamous cell carcinoma
HR: hazard ratio
i.p.: intraperitoneal
KRAS: Kirsten rat sarcoma viral oncogene homolog
lncRNA: long non‐coding RNA
LSCC: laryngeal squamous cell carcinoma
LSD1: lysine‐specific demethylase 1
LSH: lymphoid‐specific helicase
MAPK: mitogen‐activated protein kinase
MBD: methyl‐CpG binding domain
MDFI: MyoD family inhibitor
MIBP1: c‐myc intron binding protein 1
MLL3: mixed‐lineage leukemia 3
mRNA: messenger ribonucleic acid
NE: neuroendocrine
NECs: neuroendocrine carcinomas
NENs: neuroendocrine neoplasms
NETs: neuroendocrine tumors
NFI: nuclear factor I
panNET: pancreatic neuroendocrine tumor
PDAC: pancreatic ductal adenocarcinoma
PDE: phosphodiesterase
PET/CT: positron emission tomography/computed tomography
PFS: progression‐free survival
PLC: phospholipase C
p.o.: per os
PRC: Polycomb Repressor Complex
PRCRT: peptide receptor chemoradionuclide therapy
PTP: protein tyrosine phosphatase
RB1: Retinoblastoma 1
RISC: RNA‐induced silencing complex
RNA: ribonucleic acid
RPT: radiopharmaceutical therapy
SAHA: suberoylanilide hydroxamic acid
s.c.: subcutaneous
SCLC: small cell lung cancer
SD: stable disease
SEF‐2: serum‐element‐binding factor 2
SI‐NETs: small intestinal neuroendocrine tumors
SSAs: somatostatin analogs
SST: somatostatin
SSTR: somatostatin receptor
SSTR1: somatostatin receptor type 1
SSTR2: somatostatin receptor type 2
SSTR3: somatostatin receptor type 3
SSTR4: somatostatin receptor type 4
SSTR5: somatostatin receptor type 5
SUV: standardized uptake value
TF: transcription factor
TNBC: triple‐negative breast cancer
TP53: tumor protein p53
TSS: transcription start site
UTR: untranslated region
VEGF: vascular endothelial growth factor
VPA: valproic acid

## 1 Introduction

The somatostatin receptor (SSTR) system represents a central inhibitory signaling pathway regulating cell proliferation, hormone secretion, and angiogenesis across multiple organ systems [1, 2]. Its biological effects are mediated through five G protein–coupled receptor subtypes (SSTR1–5), which exhibit distinct tissue distribution and signaling properties [3, 4].

Among these subtypes, SSTR2 has the greatest clinical relevance in oncology. Its surface expression underlies both the antiproliferative efficacy of somatostatin analogs (SSAs), as demonstrated in the PROMID trial [5] and CLARINET trial [6], and targeted radionuclide delivery via radiopharmaceutical therapy (RPT) with [^177^Lu]Lu‐DOTATATE, validated in the NETTER‐1 and NETTER‐2 trials [7, 8, 9, 10]. Both therapeutic strategies depend on sufficient SSTR2 surface expression, while uptake on SSTR‐directed PET/CT provides a functional readout of receptor availability.

However, approximately 20%–30% of gastroenteropancreatic neuroendocrine tumors (GEP‐NETs) and the majority of poorly differentiated neuroendocrine carcinomas (NECs) exhibit insufficient SSTR2 expression, limiting access to receptor‐targeted therapies [11, 12]. This raises the question of whether receptor loss reflects irreversible genetic alterations or reversible epigenetic modifications.

At the biological level, physiological SSTR expression is closely linked to neuroendocrine differentiation programs in well‐differentiated NETs [13, 14]. Loss of receptor expression during progression toward poorly differentiated NEC likely reflects a combination of lineage plasticity and epigenetic repression [14, 15, 16]. Distinguishing between these processes may be important for understanding therapeutic resistance and the biological rationale for epigenetic priming.

Accumulating evidence indicates that epigenetic mechanisms play a central role in the reversible regulation of SSTR expression. Early studies identified a CpG island‐regulated promoter region of *SSTR2* controlled by DNA methylation and histone acetylation [17], and subsequent work has revealed a multilayered regulatory network involving chromatin remodeling and non‐coding RNAs. These insights have led to the concept of epigenetic priming, in which DNA methyltransferase inhibitors (DNMTi) and histone deacetylase inhibitors (HDACi) restore functional receptor expression and re‐sensitize tumors to receptor‐targeted therapies, with emerging evidence from early clinical studies supporting its translational potential [18, 19, 20].

In this review, we synthesize current knowledge on the epigenetic regulation of all five SSTR subtypes, with particular emphasis on SSTR2 and SSTR5. We examine promoter architecture, the relationship between epigenetic modifications and clinical phenotypes, and critically evaluate preclinical and clinical strategies aimed at restoring receptor expression in neuroendocrine and non‐neuroendocrine malignancies.

## 2 Molecular Mechanisms of SSTR Regulation

### 2.1 Physiological and Lineage‐Specific Regulation of SSTR Expression

Physiological SSTR2 expression is closely associated with neuroendocrine differentiation and is typically retained in well‐differentiated NETs [13, 14]. Consistent with this lineage‐associated phenotype, SSTR2 expression frequently parallels established neuroendocrine markers including chromogranin A and synaptophysin [21, 22]. In contrast, poorly differentiated NECs commonly exhibit reduced SSTR2 expression together with loss of differentiated neuroendocrine features and acquisition of proliferative molecular programs involving *TP53* and *RB1* alterations [14, 23].

The transcriptional mechanisms governing lineage‐specific SSTR2 expression remain incompletely defined but likely reflect coordinated regulation of neuroendocrine secretory, vesicular, and receptor‐signaling pathways. Preservation of neuroendocrine differentiation is associated with maintenance of transcriptional programs and chromatin accessibility at genes involved in hormone secretion and receptor signaling, whereas progression toward poorly differentiated phenotypes is accompanied by widespread transcriptional reprogramming and lineage plasticity [14, 23, 24]. Such transcriptional reprogramming provides an additional mechanism contributing to reduced SSTR2 expression independently of direct promoter methylation.

These lineage‐associated differences also help explain why many epithelial malignancies exhibit low baseline SSTR2 expression. In non‐neuroendocrine tumors, the somatostatin signaling axis is generally not a core component of the cellular differentiation program, and receptor expression is therefore more susceptible to epigenetic silencing during tumor progression [25, 26, 27, 28, 29]. Nevertheless, residual or inducible SSTR2 expression in selected non‐neuroendocrine tumors suggests that lineage restriction is not absolute and that receptor repression may remain partially reversible in certain contexts.

Together, these observations support a model in which SSTR2 expression reflects both cellular lineage identity and epigenetic state. Distinguishing between loss of receptor expression driven by dedifferentiation and potentially reversible epigenetic repression is therefore important for understanding therapeutic resistance and the rationale for epigenetic priming strategies.

### 2.2 DNA Methylation at the *SSTR2* Locus

Epigenetic silencing of *SSTR2* was first identified in pancreatic cancer cell lines that retained the gene but lacked detectable receptor expression. In these models, transcription is regulated by an upstream promoter located approximately 3.8 kb upstream of the canonical transcription start site, embedded within a CpG island whose methylation status determines transcriptional activity [17]. Pharmacological demethylation with decitabine, particularly in combination with histone deacetylase inhibition, restored *SSTR2* mRNA expression, establishing the epigenetic basis and reversibility of receptor silencing (Figure 1A).

**FIGURE 1:**
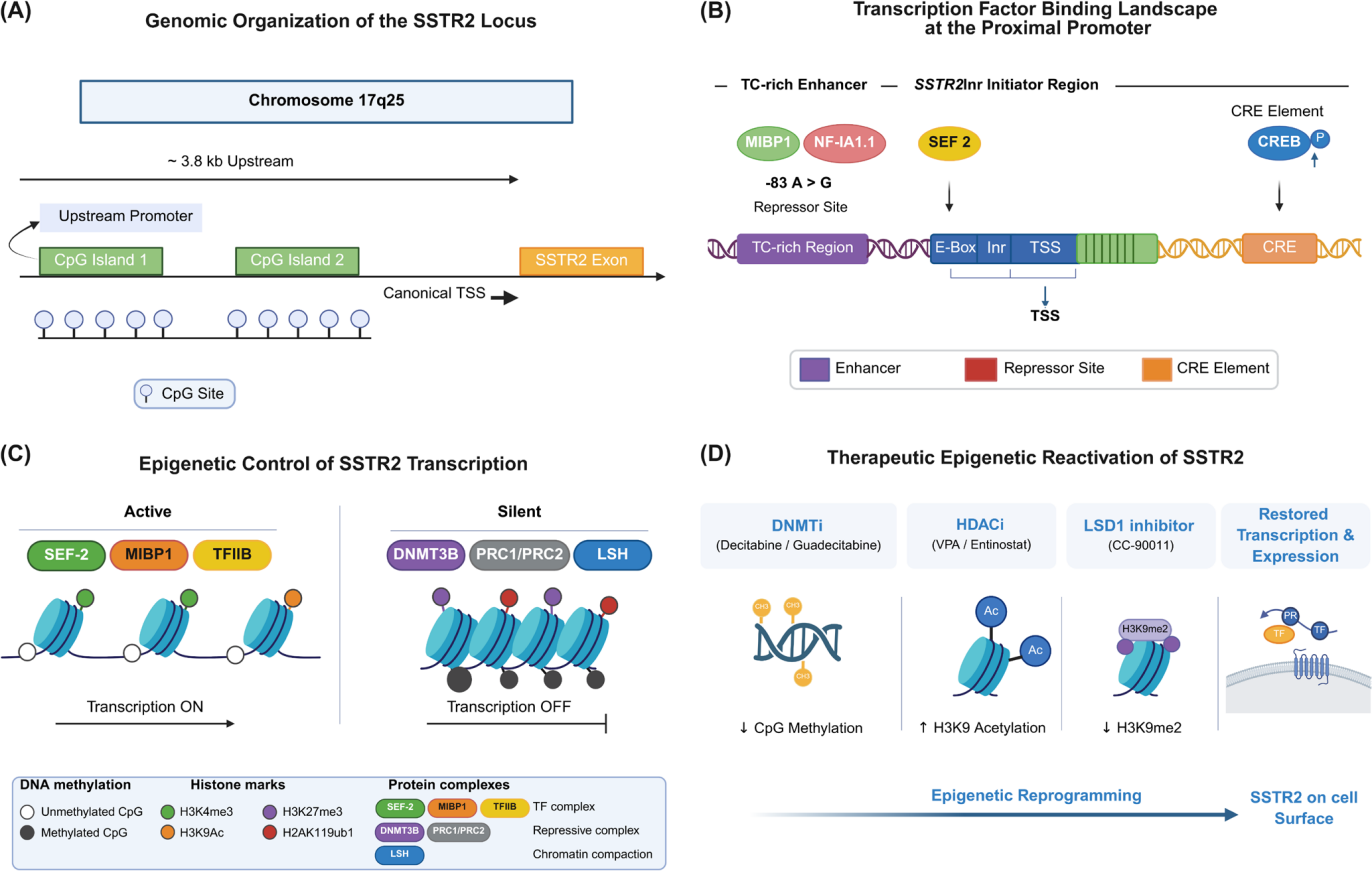
Molecular architecture of SSTR2 epigenetic regulation. (A) Schematic of the human *SSTR2* gene locus (17q25) showing the two CpG islands ~3.8 kb upstream of the canonical TSS adapted from Torrisani et al., with annotated CpG dinucleotide positions and the upstream promoter embedded within CpG island 1. (B) Transcription factor binding landscape at the proximal promoter: *SSTR2*inr initiator with SEF‐2/E‐box, TC‐rich enhancer with MIBP1, NF‐IA1.1 repressor site created by the −83 A → G polymorphism, and the CRE element with CREB binding. (C) Epigenetic states contrasted: Left—active transcription with unmethylated CpGs, H3K9Ac, H3K4me3, and accessible chromatin allowing SEF‐2/MIBP1/TFIIB assembly; Right—silenced state with methylated CpGs, DNMT3B recruitment, PRC1/PRC2‐deposited H3K27me3 and H2AK119ub1, LSH‐mediated nucleosome remodeling, and deacetylated H3K9. (D) Pharmacological reversal: DNMTi (decitabine/guadecitabine) removes CpG methylation; HDACi (VPA/entinostat) restores H3K9Ac; LSD1 inhibitors (CC‐90011) remove H3K9me2; combined treatment restores transcription factor access and mRNA expression. Arrows indicate the flow from epigenetic modification to functional SSTR2 protein on the cell surface.

The proximal 5′‐flanking region of *SSTR2* contains multiple regulatory elements that coordinate transcriptional control. Early characterization of approximately 1.5 kb upstream of the transcription start site identified GC‐rich sequences, including Sp1 binding sites and an initiator element, consistent with features of TATA‐less promoters [30]. Subsequent studies defined a transcription factor network centered on a novel initiator element (*SSTR2inr*), which is regulated by the basic helix–loop–helix factor SEF‐2 through binding to an E‐box motif and recruitment of the general transcription factor TFIIB [31]. The zinc finger protein MIBP1 binds an adjacent TC‐rich enhancer and physically interacts with SEF‐2 to synergistically activate transcription [32]. CpG methylation within or proximal to these binding sites prevents activator access, providing a direct mechanistic link between promoter methylation and transcriptional silencing (Figure 1B; Table 2).

Additional regulatory complexity arises from genetic variation within the promoter region. A single‐nucleotide polymorphism at position −83 (A → G) in the proximal promoter creates a de novo binding site for the transcriptional repressor Nuclear Factor I isoform (NFI‐A1.1), although its frequency and clinical significance in neuroendocrine tumors remain poorly characterized [40]. In the pancreatic cancer cells carrying this variant, *SSTR2* transcription was reduced by 60%–70%, and the surrounding DNA methylation context was shown to modulate NFI binding affinity. This finding illustrates how genetic polymorphisms and epigenetic modifications can converge on the same regulatory region to compound transcriptional suppression. In parallel, studies in murine systems have identified alternative promoters and a functional cAMP response element (CRE) mediating inducible *SSTR2* transcription via CREB phosphorylation, indicating that receptor expression is also subject to hormonal regulation [44].

The relative contribution of epigenetic mechanisms to *SSTR2* regulation in human tissue has been evaluated in integrated analyses of primary tumors. In small intestinal neuroendocrine tumors (SI‐NETs), *SSTR2* expression correlates inversely with promoter methylation in both normal and tumor tissue (*p* = 0.006 and *p* = 0.04, respectively), whereas no consistent association with the assessed histone modifications (H3K9Ac and H3K27me3) was observed [15]. Consistent with this, tumor samples exhibit lower promoter methylation at multiple CpG sites compared to matched normal tissue, supporting DNA methylation as a dominant regulator of *SSTR2* transcription in vivo. However, histone modifications may still contribute to transcriptional regulation in a context‐dependent or permissive manner (Figure 1C).

The enzymatic machinery underlying *SSTR2* silencing involves coordinated activity of DNA methyltransferases and chromatin‐modifying complexes. Preliminary preclinical evidence from a recent bioRxiv preprint identified DNMT3B as the principal de novo methyltransferase responsible for promoter methylation, with lesser contributions from DNMT1 and DNMT3A [34]. Moreover, Polycomb Repressor Complexes 1 and 2 (PRC1/PRC2) and the chromatin remodeling factor LSH (lymphoid‐specific helicase, encoded by *HELLS*) were identified as additional components of the silencing apparatus with emerging preclinical evidence supporting *EZH2* inhibition as a potential therapeutic strategy in neuroendocrine neoplasms (NENs), although translation into clinical practice remains limited [45, 46]. LSH facilitates nucleosome repositioning to enable DNMT3B access, while PRC2 deposits the repressive H3K27me3 mark and PRC1 catalyzes H2AK119 ubiquitination, collectively establishing a self‐reinforcing silencing loop. This multilayered architecture has important therapeutic implications as disruption of a single epigenetic layer may be insufficient for sustained *SSTR2* re‐expression, supporting the rationale for combination strategies targeting multiple regulatory pathways (Table 2, chromatin remodeling section).

### 2.3 SSTR5 Regulation Through Antisense Long Non‐Coding RNA and MLL3 Recruitment

The epigenetic regulation of *SSTR5* differs fundamentally from that of *SSTR2*, primarily through the involvement of the antisense long non‐coding (lnc) RNA *SSTR5‐AS1*. Analyses of the *SSTR5/SSTR5*‐AS1 locus have identified multiple CpG‐rich regions distributed across both genes [47]. In somatotropinomas, a discordant methylation pattern has been observed, with hypomethylation of the *SSTR5* promoter region and concurrent hypermethylation of CpG islands associated with *SSTR5*‐AS1. Functional studies demonstrated that *SSTR5‐AS1* silencing reduced *SSTR5* expression and altered cellular responsiveness to the multi‐receptor SSA pasireotide [47].

Mechanistically, *SSTR5*‐AS1 physically recruits the histone methyltransferase MLL3 (*KMT2C*) to the *SSTR5* promoter, where it promotes deposition of the transcriptionally activating H3K4me3 mark [48]. Epigenetic silencing of *SSTR5*‐AS1 prevents MLL3 recruitment, leading to reduced H3K4me3 levels and loss of *SSTR5* transcription. Pharmacological treatment with DNA methyltransferase and histone deacetylase inhibitors restores expression of both *SSTR5*‐AS1 and *SSTR5*, accompanied by increased activating histone marks and reduced repressive modifications at the promoter [48]. No equivalent cis‐acting long non‐coding RNA has been identified at the *SSTR2* locus, indicating that these two clinically relevant receptor subtypes are regulated by distinct epigenetic mechanisms.

### 2.4 Epigenetic Regulation of SSTR1, SSTR3, and SSTR4

Published data on the epigenetic regulation of the remaining subtypes are comparatively limited (Table 1). Promoter methylation of *SSTR1* has been reported alongside methylation of the *SST* ligand in head and neck squamous cell carcinoma, where both appear to function as silenced tumor suppressors [49]. Evidence for epigenetic regulation of *SSTR3* is limited. Although altered expression has been reported in colorectal cancer [54], mechanistic data linking these changes to epigenetic regulation remain limited as compared to *SSTR2*. Given the distinct p53‐dependent proapoptotic signaling associated with *SSTR3* [55], its epigenetic dysregulation may have different functional consequences compared to loss of *SSTR2* expression. In contrast, *SSTR4* remains largely unexplored in the oncologic context. Its expression is predominantly restricted to neuronal tissues, and no cancer‐specific epigenetic studies have been reported to date.

**TABLE 1:** Somatostatin receptor subtypes: Biology, signaling, and epigenetic regulation.

Receptor	Gene/structure	Primary signaling	Key functions	Tumor expression	Epigenetic regulation
SSTR1	14q13; intronless; 391 aa	Gi/Go; PTP activation; ERK1/2 modulation	Antiproliferative (G1 arrest); anti‐angiogenic via VEGF	GEP‐NETs (~70%); low SSA affinity	Promoter methylation in HNSCC [49, 50]
SSTR2	17q25; 2A/2B splice variants; 369 aa	Gi; SHP‐1/2; PLC‐beta; MAPK cascade	Antisecretory; antiproliferative; proapoptotic; primary SSA/RPT target	G1/G2 NETs > 90%; decreases with grade [21, 51, 52]	CpG methylation (dominant); H3K9 deacetylation; DNMT3B; PRC1/2; miR‐185 [16, 17, 34, 35]
SSTR3	22q13.1; intronless; 418 aa	Gi; p53‐dependent apoptosis; Bax activation	Proapoptotic (unique p53 link); chemosensitivity	GEP‐NETs (~40%–70%); pituitary; breast	Limited data; altered expression reported in CRC [50, 53]
SSTR4	20p11.2; intronless; 388 aa	Gi/Go; K+ channel activation	Neuronal; anti‐inflammatory; nociception; minimal oncologic role	Low in most tumors	Virtually unstudied in cancer
SSTR5	16p13.3; multi‐exonic; 364 aa; truncated variants	Gi; PTP; MAPK; cGMP‐PDE; heterodimerizes with D2R/SSTR2	Inhibits insulin/GH; antiproliferative; pasireotide target	Somatotropinomas; corticotroph; panNETs	*SSTR5‐*AS1 lncRNA/MLL3 axis; 4 CpG islands [47, 48]

Abbreviations: aa, amino acids; CRC, colorectal cancer; D2R, dopamine receptor type 2; GEP‐NET, gastroenteropancreatic neuroendocrine tumor; GH, growth hormone; HNSCC, head and neck squamous cell carcinoma; lncRNA, long non‐coding RNA; MAPK, mitogen‐activated protein kinase; panNET, pancreatic neuroendocrine tumor; PDE, phosphodiesterase; PLC, phospholipase C; PRC, Polycomb Repressor Complex; PTP, protein tyrosine phosphatase; SSA, somatostatin analog; VEGF, vascular endothelial growth factor.

These gaps highlight the need for systematic methylation profiling across all five *SSTR* subtypes in well‐annotated patient cohorts to determine whether receptor silencing reflects a coordinated epigenetic program or subtype‐specific regulatory mechanisms.

### 2.5 Post‐Transcriptional Regulation by microRNAs

In addition to promoter‐level epigenetic control, microRNA‐mediated post‐transcriptional regulation contributes to the modulation of *SSTR2* expression (Table 2, post‐transcriptional section). The most well‐characterized interaction involves miR‐185, which directly targets the 3′‐UTR of *SSTR2* mRNA, as demonstrated by luciferase reporter assays in pituitary adenoma models, supporting a direct post‐transcriptional mechanism rather than indirect pathway modulation [41]. Overexpression of miR‐185 reduces SSTR2 protein levels, whereas inhibition of miR‐185 results in increased receptor expression. This mechanism may contribute to resistance to SSA, where receptor protein levels do not fully reflect underlying gene expression. Additional microRNAs have been implicated in *SSTR2* regulation. miR‐16‐5p has been reported to act as a positive, indirect regulator, with overexpression increasing *SSTR2* levels and enhancing the proapoptotic effects of octreotide in neuroendocrine cell models, although the intermediary targets remain undefined [42]. miR‐375 has also been suggested to influence *SSTR2* expression in corticotroph tumors, although supporting evidence remains limited [43]. Overall, the contribution of microRNAs to *SSTR2* regulation appears context‐dependent and less extensively characterized than promoter methylation. No validated microRNAs directly targeting *SSTR5* mRNA have been identified to date, further underscoring differences in regulatory architecture between receptor subtypes.

**TABLE 2:** Epigenetic and post‐transcriptional mechanisms regulating SSTR2 expression.

Regulatory level	Genomic region/element	Epigenetic mechanism	Key effectors/enzymes	Functional consequence	Biological/disease context	Key references
DNA methylation						
CpG island methylation	Two CpG islands ~3.8 kb upstream of canonical TSS (17q25); within upstream promoter	Hypermethylation of CpG dinucleotides by de novo methyltransferase; methyl‐CpG binding proteins recruited	DNMT3B (de novo); MBD proteins; methylation maintained by DNMT1	Silencing of *SSTR2*; impaired TF binding, inversely correlates with mRNA (*p* = 0.006 normal, *p* = 0.04 tumor)	Pancreatic cancer cell lines (PANC‐1, Capan‐1); panNETs (grade‐dependent: *P* = 0.000014); GEP‐NETs (86% methylated); SI‐NETs (lower methylation than normal tissue)	[15, 17, 33]
Proximal promoter methylation	CpG sites within 1.5 kb 5′‐flanking region; Sp1 sites; GC‐rich initiator	CpG methylation at TF binding sites disrupts activator binding (SEF‐2, MIBP1); may facilitate repressor recruitment	DNMT3B; competitive displacement of SEF‐2/MIBP1	Reduced basal transcription from proximal promoter; cooperates with upstream CpG island silencing	Pancreatic cancer; panNETs; studied in BON‐1, QGP‐1 cell lines	[30, 34, 35]
Pan‐cancer promoter methylation	*SSTR2* promoter CpG dinucleotides (9 CpGs in LSCC; multiple CpGs in CRC, gastric)	Progressive hypermethylation through pre‐neoplastic stages (e.g., *H. pylori* → metaplasia → dysplasia → carcinoma in stomach)	Not specified; likely DNMT3A/B in inflammation‐driven contexts	*SSTR2* silencing; independent prognostic factor in LSCC (HR = 1.127); diagnostic biomarker in CRC (AUC = 0.92 with CMTM3/MDFI)	Colorectal cancer; gastric cancer ( *H. pylori* ‐associated); laryngeal SCC; PDAC (3.1‐fold re‐induction with azacitidine)	[25, 26, 27, 29]
Histone modifications						
H3K9 acetylation (activating)	Three positions within *SSTR2* promoter (upstream CpG island region and proximal promoter)	Deacetylation by HDACs (esp. class I and class IIa HDAC4/5) closes chromatin; acetylation by HATs opens chromatin	HDAC4, HDAC5 (class IIa); class I HDACs (HDAC1/2/3); counteracted by p300/CBP HATs	H3K9Ac enrichment correlates with active *SSTR2* transcription; VPA/decitabine increases H3K9Ac at all 3 promoter positions	panNET cell lines (BON‐1, QGP‐1); cooperates with DNA methylation in silencing; LMK‐235 (HDAC4/5 selective) dose‐dependently increases SSTR2	[35, 36, 37]
H3K27me3 (repressive)	*SSTR2* promoter; 3 positions analyzed in SI‐NETs and panNET cell lines	Trimethylation by PRC2 (*EZH2*); associated with gene silencing; may recruit PRC1 for ubiquitination	PRC2 (*EZH2* catalytic subunit); PRC1 (Ring1B ubiquitin ligase)	In SI‐NETs: lower H3K27me3 in tumors vs. normal at 2/3 positions, but no correlation with SSTR2 mRNA; appears secondary to DNA methylation	SI‐NETs vs. normal tissue (primary human samples); panNET cell lines; PRC1/PRC2 confirmed as silencing factors in panNETs	[15, 34, 38]
H3K9me2 (repressive)/LSD1 demethylase	*SSTR2* promoter; studied via LSD1 inhibition	LSD1‐mediated histone demethylation marks at *SSTR2* locus	LSD1 (KDM1A); targeted by CC‐90011; synergizes with class I HDACi	Combined entinostat + CC‐90011 increases SSTR2 and [18F]SiTATE binding; suggests H3K9 demethylation and acetylation cooperate	NET cell lines (BON‐1, QGP‐1); novel combinatorial approach; 2025 data	[39]
Chromatin remodeling and polycomb						
Chromatin remodeling (LSH) + Polycomb repression	*SSTR2* promoter region; broad chromatin compaction	LSH (lymphoid‐specific helicase) remodels nucleosomes to facilitate DNMT3B access; PRC1/PRC2 deposit H2AK119ub1 and H3K27me3	LSH (*HELLS*); PRC1 (Ring1B); PRC2 (*EZH2*); DNMT3B	Cooperative chromatin repression involving LSH, DNMT3B, and PRC1/PRC2; all required for stable SSTR2 silencing	PanNETs; identified via systematic knockdown/knockout in panNET cell lines	[34]
Transcription factor—Epigenetic interplay						
Activating TFs blocked by methylation	SSTR2inr initiator element (E‐box); adjacent TC‐rich enhancer in proximal promoter	CpG methylation within/near E‐box prevents SEF‐2 binding; methylation at TC‐rich enhancer blocks MIBP1; both required for TFIIB recruitment	SEF‐2 (bHLH; binds E‐box); MIBP1 (zinc finger; binds TC‐rich enhancer); both recruit TFIIB	Loss of SEF‐2/MIBP1 binding abolishes basal transcription from SSTR2inr; methylation converts active promoter to silent state	Normal brain development (murine); pancreatic cancer cell lines; mechanism of tissue‐specific SSTR2 expression	[31, 32]
Repressor TF enabled by SNP	Position −83 in proximal promoter; A → G polymorphism creates NF‐I binding site	SNP creates de novo NF‐IA1.1 consensus; methylation status of surrounding CpGs modulates NF‐I binding affinity	NF‐IA1.1 (Nuclear Factor I); displaces activating factors	60%–70% repression of SSTR2 transcription in carriers; genetic–epigenetic convergence on same regulatory region	Pancreatic cancer cell lines; population‐level polymorphism with potential pharmacogenomic relevance for SSA response	[40]
cAMP‐responsive transcription	CRE element in murine *Sstr2* second promoter; conserved in human	Forskolin‐induced cAMP activates CREB binding to CRE; methylation of CRE‐adjacent CpGs may impair CREB access	CREB (binds CRE); PKA (phosphorylates CREB); potential epigenetic modulation of CRE accessibility	cAMP/CREB pathway provides hormone‐responsive transcriptional control; may explain tissue‐specific alternative promoter usage	Murine brain (tissue‐specific promoter usage); neuroendocrine differentiation	[30]
Post‐transcriptional regulation (miRNAs)						
miR‐185 (negative regulator)	*SSTR2* mRNA 3′‐UTR; validated direct target site	miR‐185 binds 3′‐UTR → translational repression and/or mRNA degradation; validated by luciferase assay	miR‐185 (upregulated in resistant pituitary adenomas)	miR‐185 mimics ↓SSTR2 protein; inhibitors ↑SSTR2; may explain SSA resistance in ~30% of acromegaly patients	GH‐secreting pituitary adenomas (somatotropinomas); GH3 cell line; SSA‐resistant acromegaly	[41]
miR‐16‐5p (positive indirect regulator)	Indirect mechanism; does not directly target SSTR2 3′‐UTR; likely targets a negative regulator of SSTR2	miR‐16‐5p overexpression upregulates *SSTR2* mRNA and protein; synergizes with octreotide to enhance apoptosis	miR‐16‐5p; intermediate target(s) not yet identified	Combined miR‐16‐5p + octreotide increases apoptosis beyond either alone; potential therapeutic miRNA mimic	INS‐1 neuroendocrine cells; panNET biology	[42]
miR‐375 (preliminary)	Putative *SSTR2* regulatory interaction; mechanism not fully characterized	miR‐375 manipulation alters *SSTR2* expression in murine corticotroph model; direction and mechanism under investigation	miR‐375; highly expressed in neuroendocrine cells	Potential role in corticotroph adenomas and pasireotide response	Murine pituitary corticotroph tumor cell model (AtT‐20); preliminary conference data	[43]

*Note:* This table comprehensively catalogs the multi‐layered regulatory mechanisms governing *SSTR2* expression, organized by regulatory level: DNA methylation, histone modifications, chromatin remodeling, transcription factor epigenetic interplay, and post‐transcriptional regulation by microRNAs. For each mechanism, the specific genomic region, molecular effectors, functional consequences, and biological/disease context are specified.

Abbreviations: bHLH, basic helix–loop–helix; CDA, cytidine deaminase; CRC, colorectal cancer; CRE, cAMP response element; DNMT, DNA methyltransferase; GEP‐NET, gastroenteropancreatic neuroendocrine tumor; HAT, histone acetyltransferase; HDAC, histone deacetylase; LSCC, laryngeal squamous cell carcinoma; LSD1, lysine‐specific demethylase 1; MBD, methyl‐CpG binding domain; NE, neuroendocrine; panNET, pancreatic neuroendocrine tumor; PDAC, pancreatic ductal adenocarcinoma; PRC, Polycomb Repressor Complex; RISC, RNA‐induced silencing complex; SCLC, small cell lung cancer; SD, stable disease; SI‐NET, small intestinal NET; SSA, somatostatin analog; TF, transcription factor; TNBC, triple‐negative breast cancer; TSS, transcription start site.

Taken together, *SSTR2* expression is primarily regulated by DNA methylation, with chromatin remodeling, histone modifications, and microRNAs providing additional layers of control (Table 2). In contrast, *SSTR5* is governed by a distinct mechanism involving the antisense long non‐coding RNA *SSTR5*‐AS1 and MLL3‐mediated histone activation [48]. These differences indicate that therapeutic strategies aimed at receptor re‐expression will likely need to be tailored to individual *SSTR* subtypes.

## 3 SSTR Silencing Across Tumor Entities

### 3.1 Neuroendocrine Neoplasms: Correlation With Tumor Grade and Differentiation

Across NENs, reduced SSTR2 expression during tumor progression likely reflects a combination of dedifferentiation and epigenetic repression rather than a single mechanism. The relative contribution of these processes appears to vary across tumor subtypes and grades.

A consistent finding across studies is the positive correlation between *SSTR2* promoter methylation and tumor grade in NENs. In a cohort of pancreatic NETs, methylation across multiple CpG sites was significantly associated with WHO tumor grade (*p* = 0.000014), accompanied by an inverse correlation between methylation and *SSTR2* gene expression [16]. Similarly, *SSTR2* promoter methylation has been reported in a high proportion of GEP‐NETs and is associated with reduced overall survival [33]. These findings support *SSTR2* methylation as both a biologically relevant marker and a potential prognostic indicator.

The distinction between NETs and NECs is of clear clinical relevance, as it directly determines eligibility for SSA therapy and RPT. Across NENs, *SSTR2* expression is significantly more frequent in G1/G2 NETs than in NECs (*p* < 0.0001), with positivity observed in nearly all early‐stage tumors but reduced in advanced disease, indicating a decline with tumor progression [13]. In SI‐NETs, *SSTR2* promoter methylation has been reported to be lower in tumor tissue than in adjacent normal mucosa, supporting an inverse relationship between methylation and gene expression [15]. Together with the observed correlation between methylation and tumor grade, these findings support a model in which progressive epigenetic silencing of the SSTR axis contributes to reduced receptor expression during tumor progression. Although pediatric NENs are rare, the clinical use of SSTR‐based imaging and therapy in this population has increased substantially. However, data on *SSTR2* expression remain limited; available imaging and clinical evidence suggest that receptor expression is often preserved in well‐differentiated tumors, while systematic tissue‐based and epigenetic analyses are lacking [56].

#### 3.1.1 NET G3

High‐grade neuroendocrine tumors classified as NET G3 occupy an intermediate biological position between NETs (G1/G2) and NECs. *SSTR2* expression in NET G3 is heterogeneous but consistently higher than in NECs, with reported positivity rates ranging from approximately 60%–85% compared to 10%–30% in NECs [13, 14, 57, 58, 59]. Both immunohistochemical and imaging studies demonstrate that NET G3 tumors retain SSTR expression more frequently and at higher intensity than NECs, underscoring the importance of distinguishing these entities diagnostically. This pattern is supported by cohort analyses showing that *SSTR2* expression in NET G3 more closely resembles that of G1/G2 NETs than NECs. At the molecular level, NET G3 tumors typically lack key alterations characteristic of NECs, such as *RB1* loss and *KRAS* mutations, and are defined by a Ki‐67 proliferation index > 20%, generally lower than that observed in NECs. However, partial overlap between NET G3 and NEC exists, and *SSTR2* expression remains variable across studies [23, 51, 57, 60].

Retention of *SSTR2* expression in NET G3 has direct clinical implications. Imaging studies using [^68^Ga]Ga‐DOTATATE PET demonstrate receptor positivity in approximately 80%–88% of cases, a rate comparable to G1/G2 NETs, although often with lower intensity [61, 62]. However, receptor positivity alone may not fully predict therapeutic efficacy, as the intensity and homogeneity of SSTR expression also influence responses to receptor‐targeted therapies. Higher [^68^Ga]Ga‐DOTATATE uptake generally correlates with improved RPT response, reflecting greater receptor density and radioligand delivery to tumor tissue [63]. Conversely, heterogeneous or low‐intensity uptake may indicate subclonal variation in SSTR2 expression and has been associated with reduced therapeutic benefit despite nominal receptor positivity [64, 65]. Similar principles likely apply to SSA therapy, where receptor density influences downstream signaling and antiproliferative efficacy. These observations suggest that both the presence and functional magnitude of SSTR2 expression are clinically relevant in NET G3 tumors. This is supported by the NETTER‐2 trial, which included patients with SSTR‐positive, well‐differentiated GEP‐NETs with Ki‐67 indices up to 55%, encompassing high G2 and G3 tumors. Treatment with [^177^Lu]Lu‐DOTATATE resulted in improved progression‐free survival and objective response rates compared to high‐dose octreotide, with subgroup analyses suggesting comparable or higher response rates in NET G3 relative to G2 tumors (48.1% vs. 40.4%) [8].

Despite these findings, the relationship between *SSTR2* promoter methylation and tumor grade in NET G3 remains insufficiently defined. Available data suggest a positive association between methylation and tumor grade, but the small number of G3 cases limits interpretation [66]. Further studies are needed to clarify the role of epigenetic regulation of *SSTR2* in this subgroup.

#### 3.1.2 Dynamic Regulation of SSTR2 Under Therapeutic Pressure

*SSTR2* expression is dynamic and may change during therapy. Several mechanisms may contribute to apparent loss of receptor expression, including receptor internalization, clonal selection, tumor dedifferentiation, and potentially epigenetic silencing.

Receptor internalization following ligand binding is a well‐characterized feature of G protein‐coupled receptors. *SSTR2* undergoes β‐arrestin–mediated internalization upon agonist stimulation but is rapidly recycled to the plasma membrane rather than degraded. Consistent with this, prolonged exposure to SSAs does not significantly reduce *SSTR2* gene expression in vitro, indicating that receptor trafficking alone is unlikely to account for sustained loss of expression [67, 68, 69].

Epigenetic silencing represents a plausible mechanism of acquired resistance under therapeutic pressure. Cross‐sectional studies show that *SSTR2* promoter methylation correlates with reduced receptor expression and higher tumor grade [15, 16, 33]. However, it remains unclear whether therapies such as SSA or RPT actively induce de novo methylation, as longitudinal analyses of paired tumor samples are lacking.

Intratumoral heterogeneity provides an alternative explanation. Quantitative imaging studies demonstrate variability in *SSTR2* expression both within and between lesions, suggesting that receptor‐targeted therapies may preferentially eliminate receptor‐high cells while allowing expansion of pre‐existing receptor‐low clones [64, 65].

Histologic transformation from NET to NEC has also been described following therapy and is associated with reduced *SSTR2* expression, acquisition of alterations such as *TP53* and *RB1*, and poor clinical outcomes [70, 71]. These observations support a model in which tumor evolution, rather than direct receptor downregulation, contributes to loss of SSTR expression in a subset of patients.

Resistance to RPT may also occur independently of receptor expression. Functional genomic studies have identified alternative resistance mechanisms, including β‐arrestin 2 (*ARRB2*) and DNA damage response pathways, in tumors that retain *SSTR2* expression and radioligand uptake [72].

Changes in SSTR‐targeted PET uptake following therapy should be interpreted with caution, as reduced tracer uptake may reflect tumor response rather than true receptor downregulation [63]. Collectively, these observations indicate that therapeutic changes in SSTR2 expression may arise through multiple dynamic processes, including receptor trafficking, clonal selection, tumor evolution, and epigenetic remodeling.

### 3.2 Non‐Neuroendocrine Malignancies

*SSTR2* promoter methylation is not restricted to NENs but has been documented across multiple epithelial and non‐epithelial tumor types (Table 2, DNA methylation section). In colorectal cancer, combined methylation of *SSTR2* with *CMTM3* and *MDFI* achieves high diagnostic performance, with an area under the curve (AUC) of 0.92 [25]. In gastric cancer, methylation of the *SSTR2* promoter has been detected in *Helicobacter pylori* –infected mucosa and increases progressively along the metaplasia–dysplasia–carcinoma sequence, suggesting that it represents an early event in gastric carcinogenesis [26]. In laryngeal squamous cell carcinoma, methylation across multiple CpG sites within the *SSTR2* promoter is associated with disease progression and has been identified as an independent prognostic factor (HR = 1.127) [27].

Epigenetic silencing of the *SST* ligand has been described even more broadly. Hypermethylation of *SST* has been validated across multiple tumor types, including esophageal, gastric, colorectal, breast, lung, prostate, hepatocellular, and bladder cancers, supporting its potential utility as a pan‐cancer biomarker [28]. Pharmacological studies further indicate coordinated regulation of the ligand–receptor axis. Treatment with azacitidine in pancreatic ductal adenocarcinoma cells induces strong re‐expression of *SST*, together with upregulation of *SSTR2* (approximately 3‐fold), consistent with concurrent epigenetic silencing and reversibility of this pathway [29]. Similar co‐methylation patterns involving *SSTR1* and *SST* have been reported in head and neck squamous cell carcinoma [49].

Together, these findings suggest that epigenetic suppression of somatostatin signaling, involving both ligand and receptor components, may represent a broader feature of carcinogenesis rather than a neuroendocrine‐specific phenomenon. From a histogenetic perspective, this pattern is consistent with lineage‐dependent regulation of *SSTR2*, with tumors of neuroendocrine or neuroectodermal origin more likely to retain receptor expression, whereas epithelial malignancies more frequently exhibit epigenetic silencing [73, 74, 75].

### 3.3 Context‐Dependent SSTR2 Function in Small Cell Lung Cancer

An important caveat to the tumor‐suppressive model of *SSTR2* function is observed in small cell lung cancer (SCLC), where receptor expression has been detected in approximately 48% of tumors [76]. In this context, *SSTR2* appears to promote cell survival, as experimental downregulation increases apoptosis and reduces tumor growth. This context‐dependent functional divergence likely reflects the altered signaling landscape in SCLC, which is characterized by near‐universal loss of *TP53* and *RB1* [77]. These findings have direct implications for therapeutic strategies aimed at restoring *SSTR2* expression. Epigenetic reactivation of *SSTR2* in this setting could, in principle, support tumor cell survival rather than inhibit it. Careful patient selection for epigenetic priming strategies is therefore required, with consideration of downstream signaling context, particularly in high‐grade NECs where biological overlap with SCLC may exist.

In summary, epigenetic silencing of *SSTR2* is a recurrent feature across both neuroendocrine and non‐neuroendocrine malignancies. In NENs, promoter methylation correlates with tumor grade and inversely with receptor expression and patient outcomes, providing a molecular basis for differences in responsiveness to SSTR‐targeted therapies between NETs and NECs. Evidence from colorectal, gastric, laryngeal, and pancreatic ductal adenocarcinoma, together with widespread methylation of the *SST* ligand, suggests that suppression of the somatostatin signaling axis is a broader feature of carcinogenesis. However, the context‐dependent pro‐survival role of *SSTR2* in SCLC highlights that the consequences of receptor re‐expression depend on the underlying signaling environment and cannot be assumed to be uniformly tumor‐suppressive.

## 4 Pharmacological Strategies for Epigenetic SSTR Re‐Expression

The pharmacological evidence for epigenetic *SSTR2* modulation encompasses different classes of agents—DNMTi, HDACi, and their combinations tested across a range of in vitro, in vivo, and clinical settings. A comprehensive overview is compiled in Table 3; the key findings and their translational significance are discussed below.

**TABLE 3:** Pharmacological epigenetic modification of SSTR2: Preclinical and clinical evidence.

Agent(s)	Drug class	Model system	Treatment regimen	SSTR2 mRNA/protein effect	Radioligand uptake effect	Additional key findings	References
DNA methyltransferase inhibitors (DNMTi)							
Decitabine	DNMTi	BON‐1 xenografts in nude mice (in vivo)	0.002–0.2 mg/kg s.c in vivo. over 9‐days	Dose‐dependent *SSTR2* upregulation in tumor tissue	**↑**[^68^Ga]Ga‐DOTATOC uptake in vivo; improved tumor‐to‐background and tumor‐to‐kidney ratios	First in vivo demonstration of epigenetic *SSTR2* upregulation for imaging; translational proof‐of‐concept for PET enhancement	[78]
Guadecitabine (SGI‐110)	DNMTi (2nd‐gen; CDA‐resistant)	BON‐1 cells (in vitro); BON‐1 xenografts (in vivo)	5 and 10 μM in vitro; 2 mg/kg s.c. in vivo; 5 days	Significant *SSTR2* mRNA and protein upregulation	150% increase in radioligand uptake in vitro; 70% increase in vivo	No additive toxicity with [^177^Lu]Lu‐DOTATATE; improved bioavailability over decitabine; clinical methylation data: 10.5% (patients) vs. 5.2% (controls, *p* = 0.03)	[18]
Azacitidine (5‐azaC)	DNMTi	PANC‐1 (PDAC cell line)	1–5 μM; 72 h	3.1‐fold *SSTR2* induction; also > 55‐fold SST, plus *SSTR4* and *SSTR5* re‐expression	Not assessed	Coordinate reactivation of entire somatostatin signaling axis; demonstrates pan‐SSTR demethylation effect; anti‐cancer response in PDAC	[29]
ASTX727 (decitabine + cedazuridine)	Oral DNMTi (CDA inhibitor enables oral dosing)	Human patients (LANTana trial; phase Ib); NEN Ki‐67 < 55%	35 mg decitabine +100 mg cedazuridine p.o. daily; 5 days; PET day 8	4/8 patients showed SSTR2 re‐expression on PET; 2 proceeded to RPT	Measurable increase in [^68^Ga]Ga‐DOTATATE uptake in previously negative/low lesions	First‐in‐human epigenetic priming for RPT; AEs grade 1–2 (nausea); 1 grade 3 neutropenic sepsis; NCT05178693	[20, 79]
Histone deacetylase inhibitors (HDACi)							
Valproic acid (VPA)	Pan‐HDACi (class I/IIa)	BON‐1, QGP‐1, NCI‐H727 (NET); DMS53 (SCLC); clinical (8 patients)	0.1–5 mM in vitro; clinical: 500 mg of p.o. twice a day	7.4‐fold *SSTR2* mRNA increase in DMS53 (SCLC); significant upregulation in NET lines; decreases HDAC4 protein	Increased [^111^In]In‐DOTATATE uptake in NET cells; effect reversible within 7 days	Clinical pilot: Notch1 activation, SD in 4/8 patients; HDAC4 protein reduction suggests class IIa as key target; widely available/affordable	[35, 36, 80, 81]
Vorinostat (SAHA)	Pan‐HDACi	NET cell lines; pulmonary carcinoid; **clinical** (5 midgut NET pts); TNBC xenografts	1–15 μM in vitro; 25‐50 mg/kg, i.p. every 3 days; 300 mg p.o. daily clinical; 4 days	*SSTR2* upregulation across NET lines; up to 8‐fold increase in TNBC models	Clinical: 11% ↑SUV*max* in [^68^Ga]Ga‐DOTATOC across 50 liver lesions; no change in normal liver; enhanced radioligand uptake in vivo	First clinical demonstration of HDACi‐induced SSTR upregulation in patients; tumor‐selective effect (no normal tissue change); extends to non‐NEN (TNBC)	[82, 83, 84]
Romidepsin (depsipeptide)	Class I HDACi (bicyclic peptide)	Pulmonary carcinoid cells (in vitro); clinical phase II (PTCL)	1–50 nM in vitro; 13 mg/m^2^ clinical	SSTR2 surface upregulation in H727 pulmonary carcinoid cells	Increased [^68^Ga]Ga‐DOTATATE uptake and surface expression by flow cytometry	Clinical trial terminated prematurely due to cardiotoxicity concerns; limits clinical applicability as single agent	[82, 85, 86]
Panobinostat	Pan‐HDACi (hydroxamate)	BON‐1, NCI‐H727, GOT1 (NET cell lines); clinical phase II (15 low‐grade NET pts)	1–10 nM in vitro; 20 mg p.o. 3×/week clinical	Strong SSTR2 mRNA upregulation in BON‐1 and H727	Increased [^111^In]In‐DOTATATE uptake in cell lines	Clinical: 100% SD, median PFS 9.9 months; relatively well tolerated; potent but non‐selective	[50, 87]
Entinostat (MS‐275)	Class I HDACi (benzamide)	BON‐1, NCI‐H727, GOT1, QGP‐1 (NET cell lines)	0.2–1 μM; 48–72 h	Significant SSTR2 mRNA upregulation in low/medium‐expressing lines; minimal effect in high‐expressing QGP‐1	Increased [^111^In]In‐DOTATATE uptake; basis for combination with LSD1 inhibitor	Effect depends on baseline SSTR2 level (ceiling effect in high expressors); selected for combination studies with CC‐90011	[36, 39]
CI‐994 (tacedinaline)	Class I HDACi (benzamide)	QGP‐1, BON‐1, GOT‐1 cells; QGP‐1 xenografts (in vivo)	2.5–5 μmol/L 72 h in vitro, 5–10 mg/kg in vivo; daily	Significant SSTR2 upregulation in SSTR2‐low QGP‐1 model	↑[^177^Lu]Lu‐DOTATATE tumor uptake (*P* = 0.0175); tumor shrinkage vs. RPT alone (*P* < 0.0001, day 15)	Definitive preclinical proof‐of‐concept for HDACi + RPT combination; first study showing survival benefit of epigenetic priming + RPT	[16]
LMK‐235	Selective class IIa HDACi (HDAC4/5)	BON‐1, QGP‐1 (panNET cell lines)	0.02–20 μM; 48 h	Dose‐dependent SSTR2 increase	Not directly assessed with radioligand	Points to class IIa HDACs as most relevant for SSTR2; potentially fewer off‐target effects than pan‐HDACi	[37]
Mocetinostat	Class I/IV HDACi (benzamide)	BON‐1, NCI‐H727, GOT1 (NET cell lines)	84.3–171 nM, based on IC50	*SSTR2* mRNA upregulation; comparable to entinostat in potency	Increased [^111^In]In‐DOTATATE uptake	Part of systematic HDACi comparison; effect reversible within 7 days of washout	[36]
Thailandepsin‐A (TDP‐A)	Class I HDACi (natural product)	H727, UMC‐11 (pulmonary carcinoid cell lines)	2–6 nM; 48 h	Surface SSTR2 upregulation confirmed by flow cytometry	2‐fold **↑**[^68^Ga]Ga‐DOTATATE uptake; ↑receptor expression by flow cytometry	Most potent of HDACi tested in pulmonary carcinoid; also upregulates NET and ASCL1 markers	[82]
Combination strategies							
Decitabine (5‐aza‐dC) + TSA	DNMTi + pan‐HDACi	Capan‐1, PANC‐1 cell lines	2 μM decitabine +150 nM TSA; 72 h	Robust SSTR2 mRNA restoration in previously silent cells	Not assessed (predates radioligand studies)	First demonstration of SSTR2 epigenetic silencing and reversibility; identified novel upstream promoter within CpG island	[17]
Decitabine + VPA	DNMTi + HDACi	BON‐1, QGP‐1 (panNET); non‐NE controls; MPC mouse pheochromocytoma allografts	1 μM decitabine +3 mM VPA; 72 h in vitro; in vivo dosing in allografts	Synergistic SSTR2 upregulation; H3K9Ac enrichment at promoter positions; restored functional signaling	EC50 for octreotide‐mediated cAMP inhibition reduced from 60 nM to 1 nM (*p* < 0.001); enhanced radioligand binding	Receptors functionally competent (cAMP signaling); tumor‐selective: non‐NE cell lines show no radioligand uptake increase; additive with [^177^Lu]Lu‐DOTATATE in pheochromocytoma	[35, 88, 89]
VPA + Decitabine (systematic comparison)	DNMTi + HDACi	Multiple NET and non‐NET (WI‐38) cell lines	Optimized combined dosing	Superior SSTR2 upregulation vs. either agent alone across all NET lines tested	Superior [^68^Ga]Ga‐DOTATATE binding vs. monotherapy	Non‐neuroendocrine cell lines show no increase in radionuclide binding → supports tumor‐selective mechanism	[88]
Entinostat + CC‐90011	Class I HDACi + LSD1 inhibitor	BON‐1, QGP‐1 (panNET cell lines)	Entinostat 0.5–1 μM + CC‐90011 0.1–10 μM; 72 h	Potent *SSTR2* mRNA and protein upregulation; exceeds either agent alone	Enhanced [18F]SiTATE radioligand binding	Novel dual‐epigenetic approach; H3K9 demethylation (via LSD1i) cooperates with acetylation (via HDACi)	[39]
5‐FU + decitabine + TAC	Chemotherapy + DNMTi + HDACi (PRCRT concept)	BON‐1, QGP‐1 (panNET)	5‐FU ~1 μM + decitabine 0.5 μM + TAC 2.5 μM; 72 h	SSTR2 upregulation combined with radiosensitization (increased DNA damage markers)	Enhanced [^68^Ga]Ga‐DOTATOC binding in vitro	“Peptide receptor chemoradionuclide therapy” (PRCRT) concept: simultaneous SSTR2 induction + radiosensitization; dual mechanism of RPT enhancement	[90]

*Note:* This table summarizes the published data on pharmacological agents that modulate *SSTR2* expression through epigenetic mechanisms. Entries are organized by drug class (DNMTi, HDACi, combinations) and include the model system, treatment conditions, effects on *SSTR2* mRNA/protein, radioligand uptake changes, and key translational findings.

Abbreviations: AE, adverse event; CDA, cytidine deaminase; DNMTi, DNA methyltransferase inhibitor; HDACi, histone deacetylase inhibitor; i.p., intraperitoneal; LSD1, lysine‐specific demethylase 1; NE, neuroendocrine; NET, neuroendocrine tumor; NEN, neuroendocrine neoplasm; panNET, pancreatic NET; PFS, progression‐free survival; p.o., per os (oral); PRCRT, peptide receptor chemoradionuclide therapy; RPT, radiopharmaceutical therapy; s.c., subcutaneous; SCLC, small cell lung cancer; SD, stable disease; SI‐NET, small intestinal NET; TNBC, triple‐negative breast cancer.

### 4.1 DNA Methyltransferase Inhibitors

DNA methyltransferase inhibition provides the most direct strategy for reversing *SSTR2* silencing. Decitabine (5‐aza‐2′‐deoxycytidine) has been shown to restore *SSTR2* expression in both in vitro and in vivo models. In xenograft models using BON‐1 cells (human panNET cell line), treatment with decitabine results in dose‐dependent increases in [^68^Ga]Ga‐DOTATOC uptake, with improved tumor‐to‐background and tumor‐to‐kidney ratios, parameters directly relevant to imaging sensitivity and dosimetry in RPT [78]. Guadecitabine (SGI‐110), a dinucleotide prodrug of decitabine that resists degradation by cytidine deaminase and offers improved pharmacokinetics, produced a 150% increase in radioligand uptake in vitro and 70% in vivo in the same BON‐1 model, without evidence of additive toxicity when combined with [^177^Lu]Lu‐DOTATATE [18]. Pharmacological demethylation also affects the broader somatostatin signaling axis. In pancreatic ductal adenocarcinoma models, azacitidine induces re‐expression of *SST* together with upregulation of *SSTR2*, demonstrating coordinated restoration of ligand and receptor components and supporting the feasibility of epigenetic priming strategies [29] (Figure 1D).

### 4.2 Histone Deacetylase Inhibitors

The use of histone deacetylase inhibitors (HDACi) is supported by evidence that histone deacetylation at the *SSTR2* promoter contributes to transcriptional repression in conjunction with DNA methylation [35]. Pharmacological inhibition of HDACs, therefore, represents a complementary strategy to restore *SSTR2* expression. Comparative studies across multiple NET cell lines demonstrate that several HDACi, including CI‐994, entinostat, LMK‐235, mocetinostat, panobinostat, and valproic acid (VPA), increase *SSTR2* mRNA levels and enhance [^111^In]In‐DOTATATE uptake [36, 38]. However, two important limitations have been identified. First, these effects are reversible following drug withdrawal, indicating that sustained exposure or careful temporal coordination with receptor‐targeted therapies may be required [36]. Second, cells with high baseline *SSTR2* expression show limited further upregulation, suggesting a ceiling effect [36] (Table 3).

Efforts to identify the HDAC isoforms most relevant to *SSTR2* regulation have converged on class IIa enzymes, specifically HDAC4 and HDAC5. The selective class IIa inhibitor LMK‐235 increased *SSTR2* expression dose‐dependently in pancreatic NET cells [37], Similarly, VPA, which targets class I and IIa HDACs, increases *SSTR2* mRNA expression (approximately 7‐fold) in SCLC DMS53 cells and is associated with reduced HDAC4 protein levels [80]. In pulmonary carcinoid cells, multiple HDACi including thailandepsin‐A, romidepsin, suberoylanilide hydroxamic acid (SAHA), and VPA upregulated cell‐surface SSTR2 expression and produce 2‐to‐3‐fold increases in [^68^Ga]Ga‐DOTATATE uptake [82]. These findings indicate that HDAC inhibition enhances not only transcription but also functional receptor availability. Combination strategies targeting multiple epigenetic regulators may further enhance *SSTR2* re‐expression. Dual inhibition of class I HDACs and the lysine demethylase LSD1 results in greater increases in *SSTR2* expression and radioligand binding than either approach alone, supporting the concept that coordinated targeting of complementary chromatin‐modifying pathways can improve therapeutic efficacy [39]. Together, these findings support HDAC inhibition as a complementary approach to DNA methyltransferase inhibition for epigenetic priming of SSTR‐targeted therapies.

### 4.3 Combined DNMTi and HDACi Treatment

Combined inhibition of DNA methylation and histone deacetylation represents a rational strategy to reverse multilayered epigenetic silencing at the *SSTR2* locus (Table 2). Pharmacological studies demonstrate that this approach produces greater functional effects than either class of agents alone [35]. In NET cell models, combined treatment with decitabine and VPA significantly enhances receptor signaling capacity. This is reflected by a reduction in the EC50 for octreotide‐mediated cAMP inhibition from 60 nM to 1 nM (*p* < 0.001), indicating a marked increase in signaling sensitivity following receptor re‐expression [35]. This 60‐fold shift in signaling sensitivity confirmed that re‐expressed *SSTR2* is functionally coupled to downstream signaling pathways. Chromatin analyses have reported increased histone acetylation at the *SSTR2* promoter, consistent with coordinated epigenetic remodeling [35] (Figure 2A).

**FIGURE 2:**
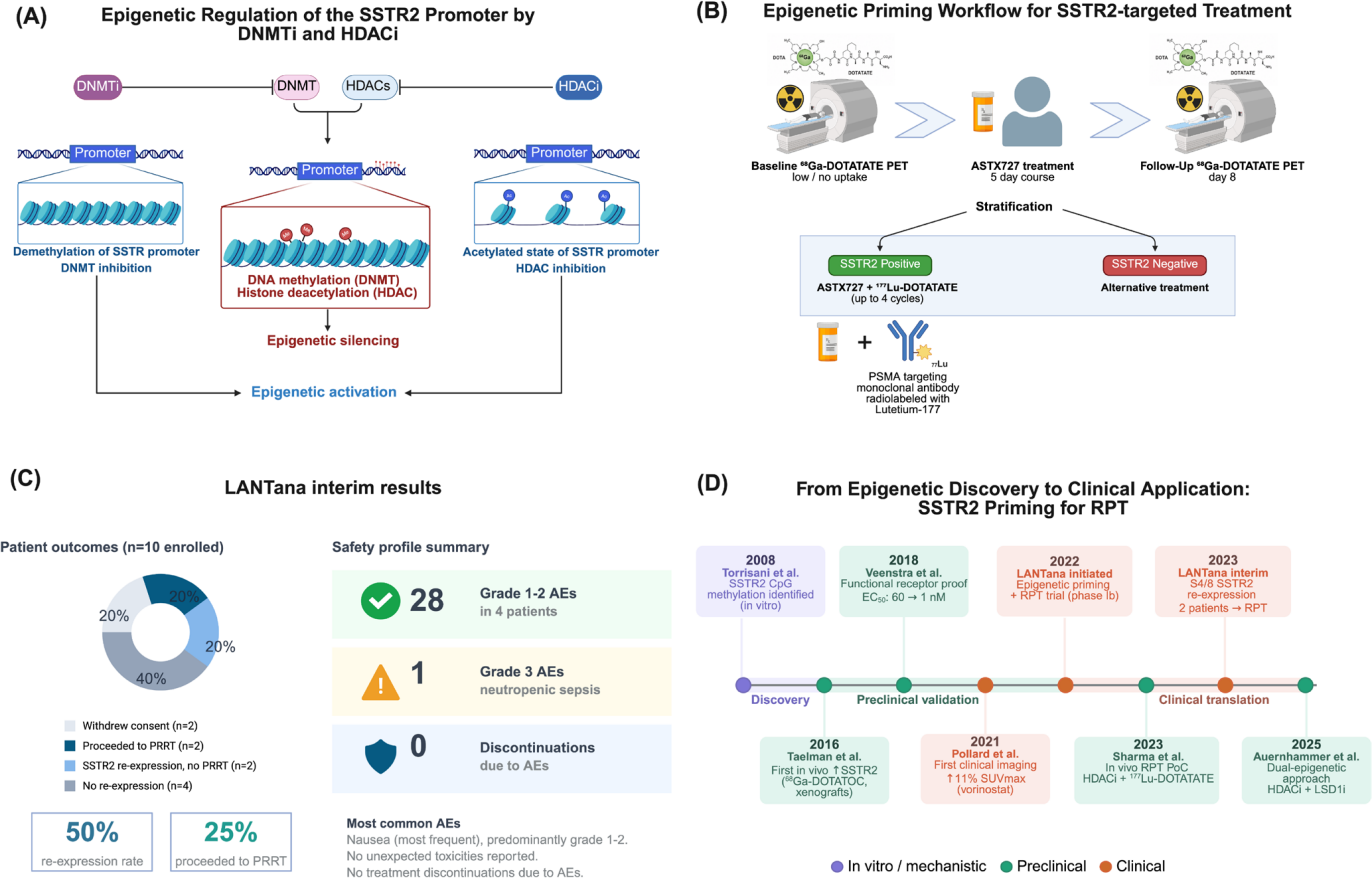
Epigenetic priming for RPT: Therapeutic concept and clinical translation. (A) Mechanisms of action: schematic showing DNMTi and HDACi targets at the SSTR2 promoter, with specific agents mapped to their molecular effects (DNMT3B inhibition by decitabine/guadecitabine/ASTX727; HDAC4/5 inhibition by VPA/LMK‐235; class I HDAC inhibition by entinostat/CI‐994; LSD1 inhibition by CC‐90011). (B) Epigenetic priming treatment sequence: Step 1: baseline [^68^Ga]Ga‐DOTATATE PET shows no/low uptake. Step 2: oral ASTX727 (decitabine 35 mg + cedazuridine 100 mg) for 5 days. Step 3: repeat PET on day 8 demonstrates SSTR2 re‐expression. Step 4: if positive, patient proceeds to combination ASTX727 + [^177^Lu]Lu‐DOTATATE (up to 4 cycles). If negative, alternative management. (C) LANTana trial (NCT05178693) interim results; pie chart of patient outcomes (4/8 re‐expression, 2/8 proceeded to RPT); safety profile summary (predominantly grade 1–2 AEs). (D) Timeline bar spanning 2008–2025: key milestones from the Torrisani discovery (2008) through Veenstra functional demonstration (2018), Sharma in vivo RPT proof‐of‐concept (2023), LANTana trial initiation (2022) and interim results (2023), to Auernhammer dual‐epigenetic approach (2025).

Consistent with this, preliminary in vitro findings showed that combined DNMTi and HDACi treatment produces greater *SSTR2* upregulation and increased [^68^Ga]Ga‐DOTATATE binding compared to single‐agent treatment across multiple NET cell lines [88]. Evidence also suggests a degree of lineage specificity, as non‐neuroendocrine cell lines exposed to the same conditions do not demonstrate increased radionuclide uptake. However, this selectivity is not absolute. In non‐neuroendocrine models such as triple‐negative breast cancer, HDAC inhibition alone has been shown to increase *SSTR2* expression at transcriptional and functional levels, with corresponding enhancement of [^68^Ga]Ga‐DOTATATE uptake in vivo [83]. These findings indicate that epigenetic priming of *SSTR2* may extend beyond classical neuroendocrine tumors, depending on the underlying chromatin context.

### 4.4 Preclinical Evidence for Epigenetic Priming Combined With RPT

Preclinical studies demonstrate that epigenetic re‐expression of *SSTR2* can enhance the efficacy of RPT. In QGP‐1 xenografts, a pancreatic NET model with low baseline *SSTR2* expression pre‐treatment with the class I HDACi CI‐994 significantly increased [^177^Lu]Lu‐DOTATATE tumor uptake (*p* = 0.0175) and, critically, produced significant tumor volume reduction compared to RPT alone (*p* < 0.0001 at day 15) [16]. Combination strategies further support this approach. Additive effects of decitabine and VPA and [^177^Lu]Lu‐DOTATATE on tumor growth delay were observed in mouse pheochromocytoma (intra‐adrenal paraganglioma) allografts [89]. Notably, the effects in the latter study were not attributable to changes in *SSTR2* promoter CpG methylation, suggesting that alternative epigenetic mechanisms possibly involving chromatin remodeling or non‐promoter regulatory elements may also contribute to treatment response. Multimodal approaches have also been explored. The concept of peptide receptor chemoradionuclide therapy (PRCRT) integrates chemotherapy with epigenetic modulation to enhance both *SSTR2* expression and radiosensitivity through increased DNA damage, providing a dual mechanism to improve treatment efficacy [90].

Collectively, these findings support several principles relevant to clinical translation. First, targeting both DNA methylation and histone deacetylation produces greater and more functionally relevant *SSTR2* re‐expression than either approach alone. Second, re‐expressed receptors are functionally active, as evidenced by enhanced downstream signaling and increased radioligand uptake. Third, epigenetic priming may exhibit partial lineage selectivity, although effects in non‐neuroendocrine models indicate that this is not absolute. Finally, and most importantly, increased *SSTR2* expression translates into improved RPT efficacy in vivo, supporting the clinical development of epigenetic priming strategies.

## 5 Clinical Translation

### 5.1 HDACi Monotherapy Trials in Neuroendocrine Tumors

Early‐phase clinical trials of HDACi monotherapy in NET patients were not designed to assess SSTR modulation but provide relevant safety and efficacy data. Treatment with VPA to NET (historically termed “low‐grade NEC”) patients has been associated with Notch1 signaling pathway activation and disease stabilization in a subset of patients [81]. Similarly, a phase II trial of panobinostat in 15 patients with G1/G2 NETs achieved stable disease in all treated patients with a median progression‐free survival of 9.9 months [87]. However, the therapeutic window remains narrow; a phase II trial of romidepsin (depsipeptide) in metastatic NET patients was terminated prematurely due to cardiac toxicity, highlighting the tolerability constraints of pan‐HDAC inhibition [91]. Collectively, these trials define the safety parameters within which epigenetic priming strategies must be developed.

### 5.2 Clinical Evidence for SSTR Upregulation by Epigenetic Agents

Direct clinical evidence that epigenetic therapy can enhance tumor *SSTR2* expression has been demonstrated using HDAC inhibition. In patients with midgut NETs, short‐term treatment with vorinostat (400 mg daily for 4 days) resulted in a statistically significant increase in [^68^Ga]Ga‐DOTATOC uptake, with an average 11% increase in SUVmax across liver metastases, while uptake in normal liver tissue remained unchanged [84]. Despite the small sample size, these findings provide in vivo evidence that epigenetic modulation can selectively increase tumor receptor expression (Table 3).

### 5.3 The LANTana Trial: Epigenetic Priming for RPT

The convergence of preclinical evidence culminated in the LANTana trial (NCT05178693), the first clinical study to directly combine a DNA demethylating agent with RPT [79]. This investigator‐initiated phase Ib trial enrolls patients with NENs (Ki‐67 < 55%) who demonstrate insufficient uptake on baseline [^68^Ga]Ga‐DOTATATE PET/CT and are therefore ineligible for RPT under standard criteria. This study specifically addresses a major clinical limitation, namely the lack of effective targeted treatment options for patients with low or absent *SSTR2* expression.

Patients receive a 5‐day course of oral ASTX727, a fixed‐dose combination of decitabine and cedazuridine (the latter inhibits cytidine deaminase to enable oral bioavailability) followed by repeat PET/CT to assess SSTR2 re‐expression. Patients demonstrating sufficient receptor upregulation proceed to combined ASTX727 and [^177^Lu]Lu‐DOTATATE therapy for up to four cycles (Figure 2B).

Preliminary interim findings from the LANTana trial indicate that *SSTR2* re‐expression can be achieved in a subset of patients, with 4 of 8 evaluable patients demonstrating increased radiotracer uptake and 2 proceeding to RPT [20] (Figure 2C). Treatment was generally well tolerated, with predominantly grade 1–2 adverse events and one reported case of grade 3 neutropenic sepsis. Although these findings are preliminary, and the trial remains in dose‐escalation, they provide proof of concept that epigenetic priming can convert patients from RPT‐ineligible to RPT‐eligible status.

The heterogeneous response observed is consistent with preclinical data and likely reflects differences in the underlying epigenetic landscape, including variability in promoter methylation and alternative silencing mechanisms beyond CpG methylation. Together with earlier clinical observations of HDAC inhibitor–induced increases in SSTR‐targeted imaging, these results support a translational framework in which epigenetic modulation enables subsequent receptor‐targeted therapy. If validated in larger cohorts, this approach could expand the population eligible for RPT, including the estimated 20%–30% of NET patients who are currently excluded due to insufficient receptor expression. Identification of predictive biomarkers to distinguish responders from non‐responders will be essential for optimizing clinical implementation.

## 6 Biomarker Potential and Future Directions

Beyond its therapeutic potential, *SSTR2* promoter methylation represents a potential predictive biomarker for RPT response. In a study of 27 patients with advanced NET treated with RPT, responders exhibited significantly lower *SSTR2* methylation than non‐responders (12.17% vs. 15.18%, *p* = 0.047), suggesting that methylation status may help identify patients most likely to benefit from this therapy [92]. Similarly, clinical studies of DNA methyltransferase inhibition demonstrate higher *SSTR2* methylation levels in NET patients than in controls (median 10.5% vs. 5.2%, *p* = 0.03), with inverse correlations between methylation, receptor expression, and radiotracer uptake [18]. Although no dedicated circulating tumor DNA assay for *SSTR2* methylation is currently available, advances in enzymatic methyl‐seq and nanopore sequencing technologies support the feasibility of developing non‐invasive liquid biopsy approaches [93]. Integration of methylation profiling with [^68^Ga]Ga‐DOTATATE PET imaging, which serves as a functional surrogate for receptor expression, may enable more precise patient stratification for both conventional and epigenetically augmented RPT.

An important extension of these concepts relates to pediatric NENs, where SSTR‐targeted imaging and therapy are increasingly used despite limited molecular characterization. The relationship between promoter methylation and receptor expression has not been systematically investigated in this population, and establishing this link will be important for future biomarker development and patient stratification.

Several emerging research directions warrant further study. Targeted epigenome editing using CRISPR‐dCas9 fused to TET family demethylases or p300 acetyltransferase domains could enable locus‐specific *SSTR2* reactivation without the genome‐wide effects inherent to systemic DNMTi or HDACi administration. The combinatorial approaches targeting multiple chromatin regulators, including dual HDAC and LSD1 inhibition, introduce a promising new axis of epigenetic intervention [39] (Table 3). In addition, modulation of the SSTR5‐AS1/MLL3 axis may provide an alternative therapeutic approach in tumors in which *SSTR5* signaling predominates [47, 48]. Further, the extension of epigenetic priming to non‐neuroendocrine malignancies in which *SSTR2* silencing has been documented including triple‐negative breast cancer [83], colorectal cancer [25], and gastric cancer [26] could substantially broaden the clinical applicability of SSTR‐targeted therapies. Finally, the systematic epigenetic characterization of *SSTR1*, *SSTR3*, and *SSTR4* across tumor entities remains an important unmet need.

## 7 Conclusion

The investigation of *SSTR* epigenetic regulation has progressed from initial mechanistic observations to clinical translation within a relatively short timeframe (Figure 2D). Several key conclusions emerge from the current evidence. First, DNA methylation at CpG islands within the *SSTR2* promoter represents a major mechanism contributing to regulation of receptor expression in human tissues, supported by integrated analyses of methylation and chromatin states in primary tumor samples [15]. Additional regulatory layers, including histone modifications, chromatin remodeling, and microRNA‐mediated mechanisms, contribute to transcriptional control but appear to act in concert with or secondary to promoter methylation (Table 2). Second, SSTR subtypes are governed by a distinct epigenetic architecture. *SSTR2* regulation centers on DNMT3B‐mediated CpG methylation with PRC1/PRC2 reinforcement, while *SSTR5* regulation depends on an antisense lncRNA (*SSTR5*‐AS1) that recruits MLL3 to deposit activating H3K4me3 marks (Table 1). In contrast, the epigenetic regulation of *SSTR1*, *SSTR3*, and *SSTR4* remains incompletely defined.

Third, and most consequentially, the pharmacological reversibility of *SSTR2* silencing has been validated across a spectrum of evidence from in vitro restoration of functional receptor signaling [35], through in vivo enhancement of radioligand uptake and RPT efficacy [16], to the first clinical demonstration of SSTR2 re‐expression in previously negative tumors in the LANTana trial [20] (Table 3). This approach addresses a clinically relevant limitation, as a substantial proportion of NET patients lack sufficient *SSTR2* expression for effective imaging or therapy. Despite these advances, several challenges remain. These include the transient effects of histone deacetylase inhibition, the need for optimized scheduling of epigenetic priming in relation to radionuclide therapy, variability in translation from preclinical models to clinical settings, and the context‐dependent role of *SSTR2* in NECs. In addition, the identification of predictive biomarkers to guide patient selection will be critical for clinical implementation. Importantly, loss of SSTR2 expression during tumor progression likely reflects a dynamic interaction between epigenetic repression, lineage plasticity, dedifferentiation, and clonal evolution rather than a single biological process.

Future studies integrating molecular profiling, functional imaging, longitudinal sampling, and prospective clinical evaluation will be required to determine whether epigenetic reactivation of *SSTR* expression can be translated into durable therapeutic benefit. Addressing these challenges will define the role of epigenetic priming within the evolving treatment landscape of NENs.

## Author Contributions

**Neeraj Kumari:** writing – original draft, writing – review and editing, conceptualization, visualization, formal analysis, investigation, supervision. **Pablo Hoffmann:** writing – original draft, investigation, writing – review and editing. **Laura Reichl:** writing – review and editing, visualization, investigation. **Maximilian Schmutz:** writing – review and editing, visualization, investigation. **Nina Fischer:** writing – review and editing. **Thi Tuong Vi Dang:** writing – review and editing. **Fulvia Ferrazzi:** writing – review and editing. **Pascal Johann:** writing – review and editing. **Michaela Kuhlen:** writing – review and editing. **Constantin Lapa:** writing – review and editing. **Rainer Claus:** conceptualization, investigation, writing – original draft, writing – review and editing, supervision, formal analysis, visualization, funding acquisition, resources.

## Conflicts of Interest

The authors declare no conflicts of interest.
